# Overexpression of α-enolase correlates with poor survival in canine mammary carcinoma

**DOI:** 10.1186/1746-6148-7-62

**Published:** 2011-10-21

**Authors:** Pei-Yi Chu, Nicholas C Hsu, Albert T Liao, Neng-Yao Shih, Ming-Feng Hou, Chen-Hsuan Liu

**Affiliations:** 1 Department of Pathology, St. Martin De Porres Hospital, No. 565, Section 2, Daya Road, Chiayi, 60069, Taiwan; 2 Department and Graduate Institute of Veterinary Medicine, School of Veterinary Medicine, National Taiwan University, No. 1, Section 4, Roosevelt Road, Taipei, 10617, Taiwan; 3 Graduate Institute of Medicine, Kaohsiung Medical University, No. 100, Shih-Chuan 1st Road, Kaohsiung, 80708, Taiwan; 4 National Institute of Cancer Research, National Health Research Institutes, No.367, Shengli Road, Tainan, 70456, Taiwan; 5 Cancer Center, Kaohsiung Medical University Hospital, No. 100, Tzyou 1st Road, Kaohsiung, 80708, Taiwan

## Abstract

**Background:**

α-Enolase (ENO1) is a key glycolytic enzyme implicated in the development of many human cancers including breast cancer. Increased expression of ENO1 has recently been reported in estrogen (ER)-positive human breast cancer patients. The present study examined the expression of ENO1 and assessed its significance in canine mammary carcinoma.

**Results:**

Immunohistochemical staining was employed to investigate the expression of ENO1 in 82 cases of canine mammary tumor (32 benign tumors and 50 carcinomas). Quantification of immunohistochemistry was carried out using Quick score and the results showed cytoplasmic ENO1 overexpression in 9 of the 50 carcinomas (18%). Overexpression of ENO1 correlated significantly with shorter cause-specific survival (P = 0.019), but was not associated with ER positivity in canine mammary carcinoma.

**Conclusions:**

Our findings suggest that overexpression of ENO1 may be used as a prognostic marker for poor outcome in canine mammary carcinoma.

## Background

Canine mammary tumor is one of the most common neoplasms in female dogs. Similar to human breast cancer, canine mammary tumor is spontaneous, and the predominant malignant histological type is carcinoma [1-3]. Previous studies have shown that estrogen and progesterone receptors (ER/PR), and epidermal growth factor receptor 2 (HER2) are expressed in canine mammary carcinoma with clinical implications similar to those in human [4-7]. It has been proposed that canine mammary carcinomas may be a suitable model for comparative oncology studies [4,5,7-9].

α-Enolase (ENO1) is a glycolytic enzyme that converts 2-phosphoglycerate into phosphoenolpyruvate in glycolysis and a multifunctional protein that play a crucial role in a variety of biological and pathophysiological processes [10]. ENO1 may act as a stress protein that promotes hypoxic tolerance in tumor cells by increasing anaerobic metabolism [11]. ENO1 may also function as a plasminogen receptor on the surface of a variety of hematopoetic, epithelial and endothelial cells [12-17]. Recently, many lines of evidence suggested that ENO1 might contribute to tumor malignancy [17-26]. Upregulation of *ENO1 *gene has been observed in several highly tumorigenic or metastatic cell lines [21,23,24] and enzymatic activities in breast cancer concluded a role of ENO1 in tumor progression [20]. A bioinformatics study using gene chips and ESTs databases further supports a correlation between ENO1 expression and tumorigenicity [18]. Increased cell-surface expression of ENO1 promotes cell transformation and invasion in non-small cell lung cancer and cancer of head and neck [19,22]. The expression of ENO1 has been also reported in pancreatic carcinoma [26] and hepatitis C virus-related hepatocellular carcinoma [25]. More recently, higher ENO1 expression was detected in ER+ breast cancer patients compared to ER- patients [27]. Patients with high ENO1 expression also had a poor prognosis with greater tumor size, poor nodal status, and a shorter disease-free survival [27].

Given the epidemiological and pathological similarity between canine mammary carcinoma and human breast cancer, and that canine mammary carcinoma may be a good animal model for the understanding of carcinogenesis and the development of treatment, the present study examined the expression of ENO1 and assessed its clinical significance in canine mammary carcinoma.

## Results

The mean age when tumors were first identified was 11.2 ± 2.9 years (range 4-18 years). The mean maximum tumor diameter was 3.8 ± 2.9 cm (range 0.2-14 cm). Of the 82 dogs, 20 had undergone ovariohysterectomy before presentation for surgical excision of the primary tumor(s). 32 cases (39%) were benign tumors and 50 cases (61%) were histologically confirmed as mammary carcinoma.

**Figure 1:**
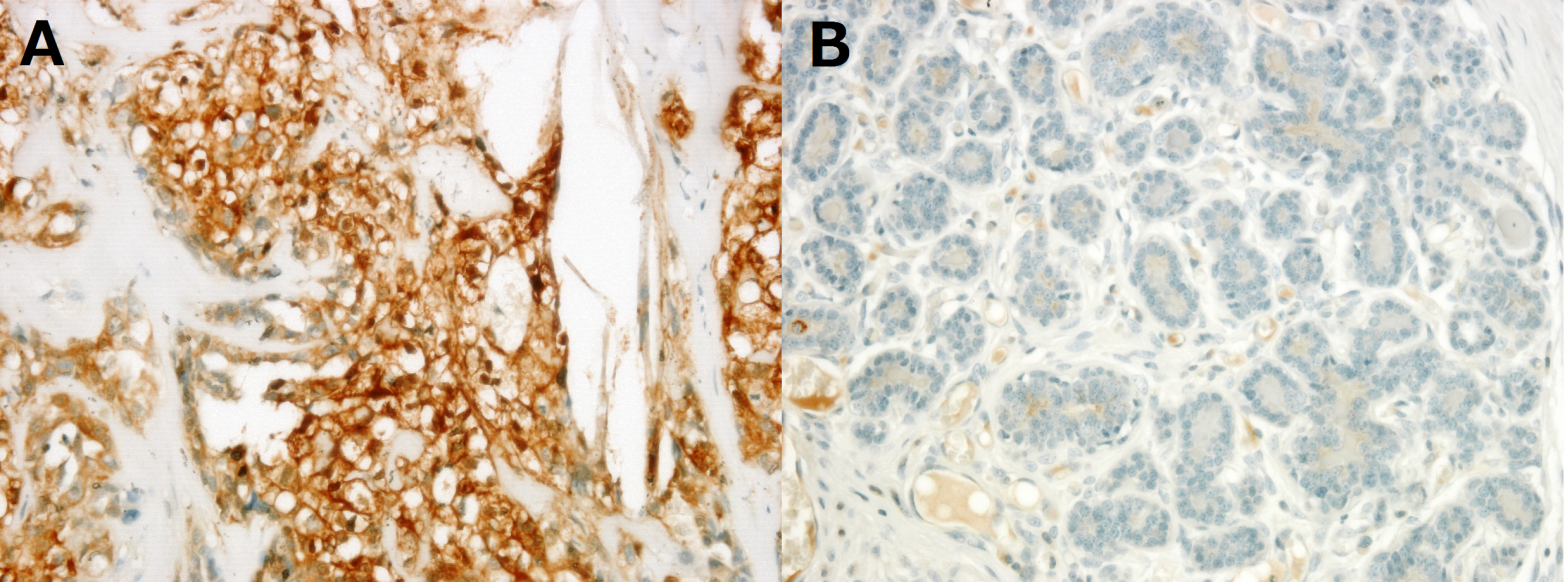
**Representative figures of the immunohistochamical staining of ENO1 in (A) canine mammary carcinoma (Quick score = 12) and (B) benign tumor (Quick score = 0)**. Carcinoma cells showed strong cytoplasmic staining of ENO1 (400×).

**Figure 2:**
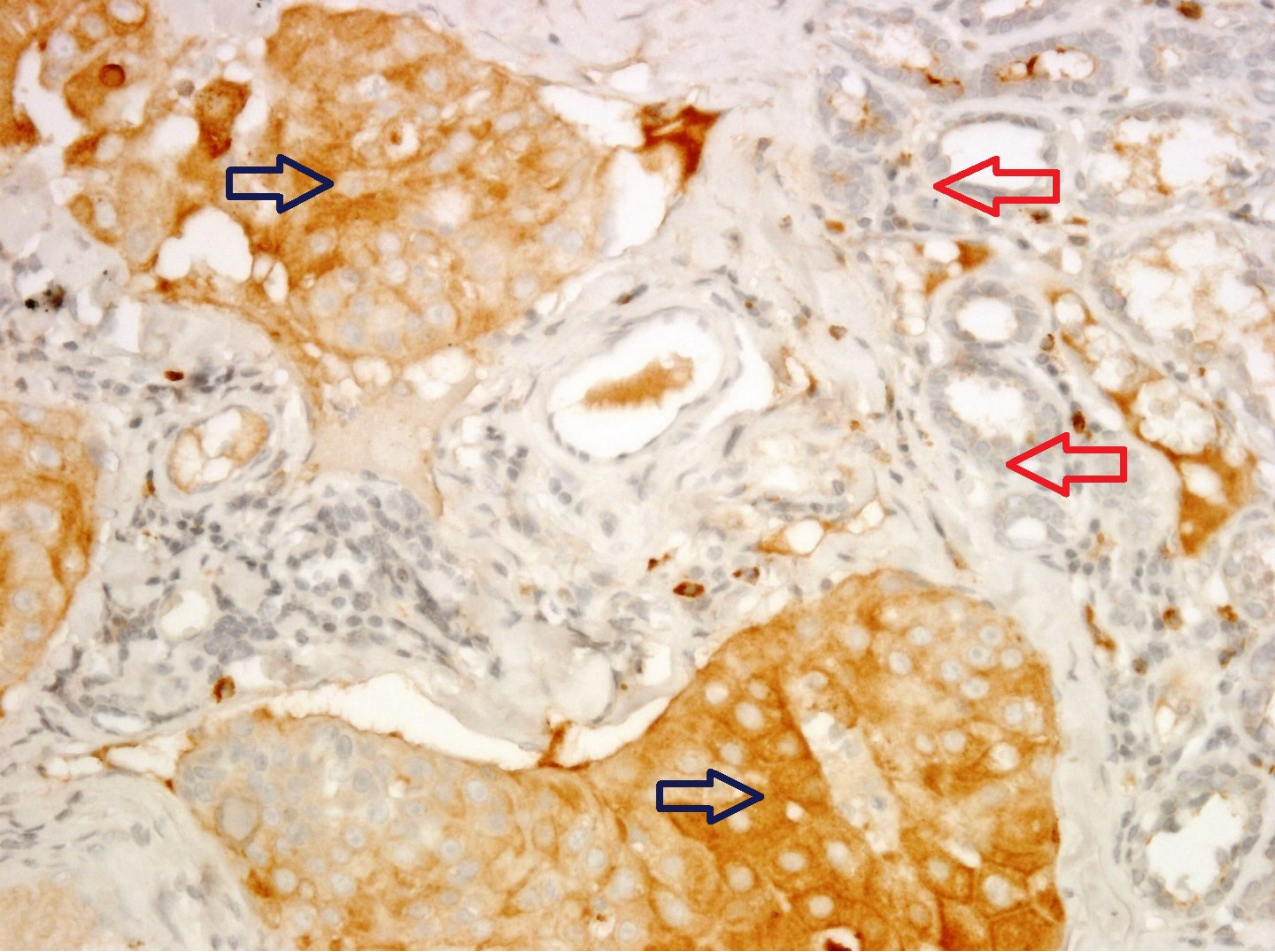
**ENO1 overexpression identified in tumor cells (blue arrow) and not adjacent non-tumor cells (red arrow) in a representative canine mammary carcinoma (400×)**.

**Figure 3:**
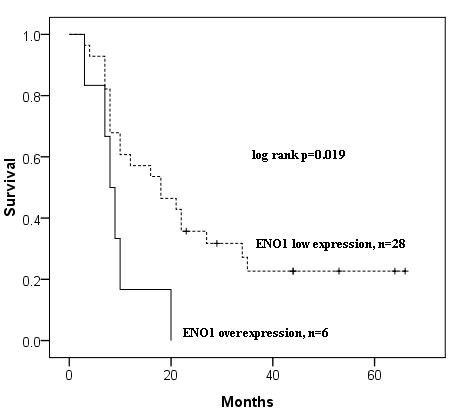
**Kaplan-Meier curves for cause-specific survival of canine mammary carcinoma patients with and without ENO1 overexpression**. Sixteen of the fifty cases lacked survival data and were excluded from the analysis.

Immunohistochemical analyses revealed that mammary carcinomas have higher expression of ENO1 as compared to benign tumors (Figure 1 and Table 1). Overexpression of ENO1 (as defined by a Quick score of 12 or greater) was only identified in 18% (9/50) of dogs with mammary carcinoma and none in the benign tumors. Moreover, ENO1 overexpression occurred preferentially in the tumor cells and not the adjacent non-tumor cells in mammary carcinoma (Figure 2 and Table 2, *P *= 0.011). The overexpression of ENO1 was not statistically associated with clinicopathologic features such as age, ovariohysterectomy, size and grade of tumor, histological classification, location of affected glands, and expression of ER, PR, and HER2. We employed the same Quick score system to quantify ER expression, although not statistically significant, a trend toward positive correlation between high expression of ER (score of ≧12) and ENO1 overexpression was found (*P *= 0.063, Table 3). Kaplan Meier survival analysis showed that cytoplasmic overexpression of ENO1 correlated significantly with shorter 5-year cause-specific survival in canine mammary carcinoma (*P *= 0.019, Figure 3). Because age is strongly related to death, control of the effect of age was accomplished by adding the mean age as a covariate to the multivariate survival analysis. The results of the age-adjusted Cox regression model showed that ENO1 overexpression retained statistical significance on cause-specific survival (*P *= 0.044, Table 4).

**Table 1:** Immunohistochemical semiquantitation of ENO1 expression with the Quick score in canine mammary tumor

*Histological classification*	Quick score				
		
	0-3	4-6	8-10	12+	total
Benign tumor	17	13	2	0	32
Carcinoma	15	15	11	9	50

**Table 2:** Overexpression of ENO1 in canine mammary tumor

ENO1 expression		Benign tumor	Carcinoma	Total	P
***Tumor part***					
Quick score	< 12	32(43.8%)	41(56.2%)	73	0.011
	≧12	0	9(100%)	9	
***Non-tumor part***					
Quick score	< 12	32(39.0%)	50(61.0%)	82	N/A
	≧12	0	0	0	

ENO1 overexpression is defined as a Quick score of ≧12

**Table 3:** Clinicopathologic characteristics of canine mammary carcinoma

	Quick score		N	P
			
	< 12	≧12		
*Age*				
< 11 years	13(81.3)	3(18.7)	16	1.000
≧11 years	27(81.8)	6(18.2)	33	
*Ovariohysterectomy*				
No	31(83.8)	6(16.2)	37	0.580
Yes	10(76.9)	3(23.1)	13	
*Tumor Size*				
T1 (< 3 cm)	15(93.8)	1(6.2)	16	0.298
T2 (3-5 cm)	13(72.2)	5(27.8)	18	
T3 (> 5 cm)	11(84.6)	2(15.4)	13	
*Grade*				
I	13(92.9)	1(7.1)	14	0.199
II/III	27(77.1)	8(22.9)	35	
*Histological classification*				
Carcinoma in benign tumor	4(80.0)	1(20.0)	5	0.566
Complex carcinoma	17(89.5)	2(10.5)	19	
Simple carcinoma	20(76.9)	6(23.1)	26	
*Location of affected gland*				
cranial	13(76.5)	4(23.5)	17	0.566
caudal	25(83.3)	5(16.7)	30	
*ER*				
Negative	22(81.5)	5(18.5)	27	1.000
Positive	19(82.6)	4(17.4)	23	
*PR*				
Negative	2(100.0)	0(0.0)	2	1.000
Positive	39(81.3)	9(18.7)	48	
*HER2 Overexpression*				
Negative	35(83.3)	7(16.7)	42	0.574
Positive	6(75.0)	2(25.0)	8	
*ER with Quick score*				
< 12	38(86.4)	6(13.6)	44	0.063
≧12	3(50.0)	3(50.0)	6	

^1^Tumor size measures maximum diameter

**Table 4:** Overexpression of ENO1 and age in the Cox regression model for predicting Cause-specific survival

Variable	Hazard ratio (95% CI)	*P*
ENO1 overexpression	2.71 (1.03-7.15)	0.044
Age (11 y.o.)	1.42 (0.57-3.58)	0.456

## Discussion

Breast cancer comparative oncology that integrates the study of canine mammary carcinoma into studies of human breast carcinoma may be uniquely positioned to take advantage of the epidemiological and clinicopathologic similarities between the two cancers of different species to improve our understanding of breast cancer biology and therapy.

Enhanced expression of ENO1 has been implicated in human tumorigenesis and also used as a diagnostic marker for human lung cancer [19,23,28-31]. ENO1 overexpression was also preferentially identified in human ER-positive breast cancer [27]. In this study, we investigated the expression and clinical relevance of ENO1 in canine mammary carcinoma. Immunohistochemical analysis revealed that overexpression of ENO1 was only detected in tumor cells of canine mammary carcinoma and significantly correlated with shorter 5-year cause-specific survival. The results of the age-adjusted Cox regression analysis further indicated that ENO1 overexpression was significantly and independently associated with shorter cause-specific survival. Unlike results from human breast cancer study, our findings suggested that ER positivity was not associated with ENO1 overexpression in canine mammary carcinoma. Although quantification of ER expression with the same Quick score system used for ENO1 revealed a trend toward positive correlation between overexpression of ER and ENO1 (*P *= 0.063). Further investigation is required to elucidate whether the molecular events that underlie ENO1 overexpression in canine mammary carcinoma is ER signaling machinery-associated or -dependent as proposed in human breast cancer [27].

The limitations of this study are primarily related to the limited number of cases and its design as a retrospective study which may make collecting complete clinical information difficult. The finding of ENO1 overexpression in the neoplastic tissue of canine mammary carcinoma and its possible role in the prognosis of this disease is clinically relevant, as ENO1 expression could be widely determined on routinely processed, paraffin-embedded tissues. Moreover, agents with ENO1 attenuation activity might provide an effective strategy for the treatment of breast cancer for both dogs and human and merit further investigation.

## Conclusions

Overexpression of ENO1 occurs in the neoplastic tissue of a subset of dogs with canine mammary carcinoma. The ENO1 overexpression may be used as a marker for poor outcome in this disease.

## Methods

### Patient Samples

Formalin-fixed, paraffin-embedded, surgically resected tissue of canine mammary tumor diagnosed between January 2003 and April 2008 were retrieved from the archives of the School of Veterinary Medicine, National Taiwan University, Taiwan. A cohort of eighty-two dogs including twenty-one Maltese, ten Yorkshire terriers, nine Shih-Tzus, seven Pomeranians, two Cocker spaniels, two French spaniels, two Bichon Frisé, one poodle, one German shepherd dog, one Shiba, one Beagle, one Labrador Retriever and twenty-four mongrels with canine mammary tumor were analyzed in this study. Archived hematoxylin and eosin (HE) sections from samples fixed in 10% buffered formalin and embedded in paraffin wax were reviewed to assess the diagnoses. The tumors were diagnosed according to the World Health Organization (WHO) Histological Classification of Mammary Tumors of the Dog and the Cat [32]. Tumor size was classified according to the WHO Clinical Staging System TNM as T1 (< 3 cm maximum diameter), T2 (3-5 cm maximum diameter) and T3 (> 5 cm maximum diameter) [33]. The largest one was used as the basis for classification in cases of more than one tumor. Histological grading was performed on HE-stained sections and graded by a scheme based on tubule formation, nuclear pleomorphism, and mitotic counts [34,35]. Each feature was scored 1 to 3 points. The scores were then added to obtain the tumor grade. Final scores of 3-5 points, well-differentiated, were designated grade I; scores of 6 and 7 points, moderately differentiated carcinoma, were designated grade II; and scores of 8 and 9 points, poorly differentiated carcinoma, were designated grade III.

### Immunoblotting

To detect endogenous the ENO1 protein and examine antibody specificity, canine mammary carcinoma cell line CF41 (ATCC, Rockville, MD, USA) was lysed in PBS/TDS lysis buffer (10 mM Na_2_HPO_4_/150 mM NaCl/1% Triton X-100/0.5% sodium deoxycholate/0.1% SDS/10 mM NaF, pH 7.25) containing the protease inhibitor cocktail (Calbiochem, San Diego, CA). The protein concentrations of the lysates were determined using the BCA protein assay kit (Pierce, Rockford, IL). The lysates were resolved in a 10% SDS-containing polyacrylamide gel, blotted on a nitrocellulose membrane, and probed with anti-ENO1 antibody (clone 8G8, Abnova Co., Taipei, Taiwan) in 1:2000 dilution, or with pre-immunized mouse total IgG. The immunocomplex was detected by the goat anti-mouse IgG antibody conjugated with horseradish peroxidase (Jackson ImmunoResearch Labs, West Grove, PA) and visualized by SuperSignal chemiluminescence (Pierce, Rockford, IL). β-Actin (Sigma, St. Louis, MO) was the loading control.

### Immunohistochemistry

Paraffin-embedded canine mammary tumor tissue sections (4-μm) on poly-1-lysine-coated slides were first de-waxed in xylene and re-hydrated through graded alcohols, followed by a rinse using 10 mM Tris-HCl (pH 7.4) and 150 mM sodium chloride, then treated with 3% hydrogen peroxide for 5 min. Slides were incubated with 1:2000 dilution of anti-ENO1 antibody for 1 hour at room temperature, then thoroughly washed three times with PBS. Bound antibodies were detected using the LSAB+ kit (DAKO, Carpinteria, CA). The slides were then counterstained with Gill Hematoxylin Solution II (MERCK, Darmstadt, Germany). Paraffin-embedded sections of human breast cancer cells of homogeneous ENO1 immunophenotype were included as positive controls. Negative controls had the primary antibody omitted and replaced by pre-immunized mouse total IgG. Quantification of immunohistochemistry was carried out using Quick score which multiply the staining intensity by the percentage of positive cells [36-38]. The intensity of staining was scored as 0, 1, 2, and 3 standing for negative, weak, moderate, and strong staining, respectively. The percentage of tumor cells staining positively was scored as follows: 0 = 0%, 1 = 1-20%, 2 = 21-40%, 3 = 41-60%, 4 = 61-80, and 5 = 81-100%, compared with the total of tumor cells.

Immunohistochemistry was also performed in parallel as described above with mouse monoclonal antibodies for ER (clone 1D5, 1:35 dilution, Dako, Denmark), PR (clone SP2, 1:200 dilution, Thermo Scientific, Fremont, CA), and HER2 (A0485, 1:400 dilution, Dako, Denmark). ER and PR immunoreactivity was considered positive when more than 10% of the neoplastic cells expressed this marker [4]. The American Society of Clinical Oncology/College of American Pathologists guidelines were used to evaluate HER2 expression (0 = no staining or membrane staining in fewer than 10% of tumor cells; 1+ = faint, barely perceptible membrane staining in more than 10% of tumor cells; 2+ = weak to moderate complete membrane staining observed in more than 10% of tumor cells or strong complete membrane staining in less than 30% of tumor cells; 3+ = strong and complete membrane staining in more than 30% tumor cells)[39]. In this study, overexpression of HER2 was defined as a score of 3+.

Histological grading and immunohistochemical results were evaluated by two investigators (veterinary pathologists) scoring independently. Conflicting scores were resolved at a double-headed microscope.

### Statistical Analysis

Overexpression of ENO1 was defined as a Quick score of 12 or greater on the scale of 0 to 15. Patient data were obtained from medical records. Correlations of ENO1 and clinicopathologic parameters of canine mammary carcinoma were examined by Pearson's chi-square test. Survival rate was calculated using Kaplan-Meier analysis and compared by the Cochran-Mantel-Haenszel test (log-rank test). Cox proportional hazards regression model was used to control the effect of age on survival. Cause-specific survival was defined as the time between date of diagnosis and date of cancer-related death. Follow-up was obtained by telephone call up to August 2010. If a patient had died, the information regarding the cause of death was obtained by contact with the veterinarian. Subjects still alive at the end of the study were censored at the date of last follow-up. Cases that lacked survival data were excluded from the analysis. A *P *value of less than 0.05 was considered to indicate statistical significance.

## Authors' contributions

PYC drafted the manuscript, NCH performed the statistical analysis, ATL carried out the immunohistochemical staining, NYS, MFH, and CHL designed the study. All authors read and approved the final manuscript.
